# Isolation, identification and characterization of *Burkholderia pseudomallei* from soil of coastal region of India

**DOI:** 10.1186/2193-1801-3-438

**Published:** 2014-08-16

**Authors:** Archana Prakash, Duraipandian Thavaselvam, Ashu Kumar, Ajith Kumar, Sonia Arora, Sapana Tiwari, Anita Barua, Kannusamy Sathyaseelan

**Affiliations:** Division of Microbiology, Defence Research & Development Establishment, Jhansi Road, Gwalior, 474 002 India; Centre for Advanced Studies in Marine Biology, Annamalai University, Parangipettai, Tamil Nadu India

**Keywords:** *Burkholderia pseudomallei*, Ashdown agar, Melioidosis, Parangipettai, Soil isolate

## Abstract

Melioidosis is an emerging infectious disease caused by a free living soil dwelling Gram-negative bacterium *Burkholderia pseudomallei*. The disease is endemic to most parts of Southeast Asia and northern Australia and the organism has been isolated from moist soil and water. In India clinical cases are recently reported from the states of Tamilnadu, Kerala, Karnataka, Maharashtra, Orissa, Assam, West Bengal, Pondicherry and Tripura. This study is aimed to confirm the prevalence of this important bacterial species in soil samples collected from coastal areas of Tamilnadu*.* Forty five soil samples from five different sites were collected from Parangipettai, Tamilnadu and screened for the presence of *B. pseudomallei*. The study confirmed 4 isolates as *B. pseudomallei* with the help of conventional bacteriological methods and molecular methods that include; 16S rDNA sequencing, *B. pseudomallei* specific PCR, *fli*C gene RFLP and MALDI-TOF mass spectrometry based bacterial identification. This study reveals the prevalence and distribution of *B. pseudomallei* in the soil environment in coastal areas of southern India and further necessitates studies from other parts of the country. It will also be helpful to understand the distribution of *B. pseudomallei* and to access its epidemiological importance.

## Introduction

Melioidosis is caused by soil dwelling Gram-negative bacterium *Burkholderia pseudomallei* and is an emerging infectious disease in India. The disease is mainly endemic in Southeast Asia and northern Australia with highest number of melioidosis cases reported from Thailand. The global distribution boundaries of melioidosis continue to expand well beyond the traditionally recognized endemic regions (Currie et al., 2008). In India, clinical cases have been reported from states of Tamilnadu, Kerala, Karnataka, Maharashtra, Orissa, Assam, West Bengal, Pondicherry and Tripura. *Burkholderia pseudomallei* has been isolated from clinical samples like blood, sputum, pus, urine, synovial, peritoneal and pericardial fluids mostly from tertiary care hospitals located at Vellore, Tamil Nadu and Mangalore, Karnataka (Raghavan et al., 1991; Kavitha et al., 2008). The true incidence of melioidosis is not known in India and recently larger number*s* of cases have been reported from the western coastal areas (Vidyalakshmi et al., 2007). Melioidosis is referred to as “a great imitator” because of its wide spectrum of clinical presentations, ranging from mild subclinical infection to fatal septicaemia that can be chronic, localized or disseminated. The infection occurs through inhalation, or skin abrasions that come in contact with contaminated soil or water. Diabetes is the most common risk factor that is associated with the disease and other risk factors include thalassaemia, alcoholism and renal impairment. Isolation of the organism from soil is required to define the epidemiology and distribution of *B. pseudomallei,* and the associated risk to humans and livestock (White, 2003; Leelarasamee, 2004). Earlier studies have shown the presence of *B. pseudomallei* in the environment based on culture of soil and water from different geographic regions, particularly from Southeast Asia and northern Australia (Strauss et al., 1969). The description of *B. thailandensis,* a non-virulent but closely related species present in the soil, has made the isolation and characterization of *B. pseudomallei* from soil very challenging (Brett et al., 1998). This species has similar colony morphology characteristics to *B. pseudomallei* on solid media and biochemical and molecular techniques are needed to distinguish between them. The isolation of *B. pseudomallei* from different soil depths and during different seasons of the year has been studied, and quantitative culture of *B. pseudomallei* from soil samples has also been done in many countries previously (Smith et al., 1995; Brook et al., 1997). Recently a review for the global presence and distribution of *B. pseudomallei* clearly indicates that isolation of this species from soil has not been reported from India, despite its isolation from human cases (Limmathurotsakul et al., 2013). The present study was undertaken to attempt the isolation of *B. pseudomallei* from the coastal rice cultivating areas of Tamil Nadu, India to confirm the identity of isolates by conventional and molecular methods.

## Materials and methods

### Study site and collection of samples

**Figure 1:**
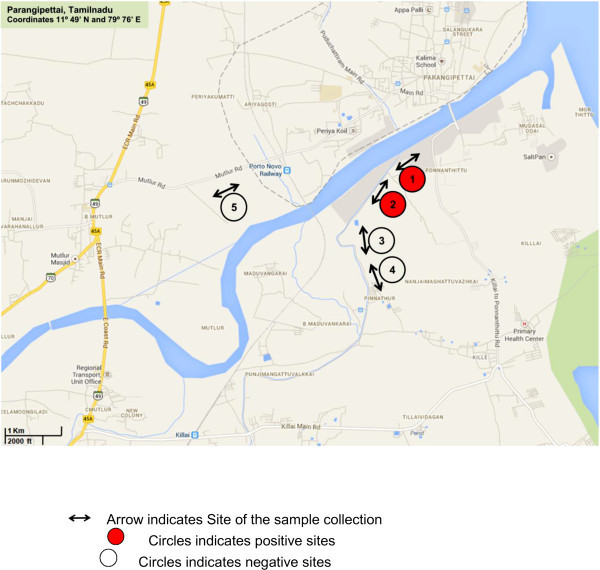
**Soil sampling and five study sites of Parangipettai, Tamilnadu, India indicated by arrow as shown in map.**

The southeast coast of Parangipettai, District Cuddalore, Tamilnadu, India (11° 49′ N and 79° 76′ E) was selected as the sampling site for this study. Parangipettai is 30.3 km from the main city of Cuddalore and 183 km from Chennai. The annual average rainfall of this area is approx. 945.0 mm, mean relative humidity 57% and average temperature range between 28°C to 40°C in summer and 18°C to 26°C in the short lived winter season. Five paddy fields were chosen as study sites and the sampling was done just after the rainy season from July to Sept. 2010 at a depth of 25 to 30 cm. Among the five paddy fields sites, sites1 and 2 belong to Ponnanthittu village; sites 3 and 4 belong to Pinnathur village situated on the left side of the Vellar river and site 5 is situated right side of the Vellar river, near to Mutlur road (Figure 1). Ten sampling points were selected from site1 to site 4 and five sampling points from site 5. Soil samples were collected at 100 m distance intervals from each other in a straight line. Approximately 100 gm of moist soil were collected from each sampling points and immediately placed into a sterile 50 ml conical centrifuge tubes. The tubes were sealed to avoid contamination and transported to the laboratory for further processing.

### Soil processing and isolation

**Figure 2:**
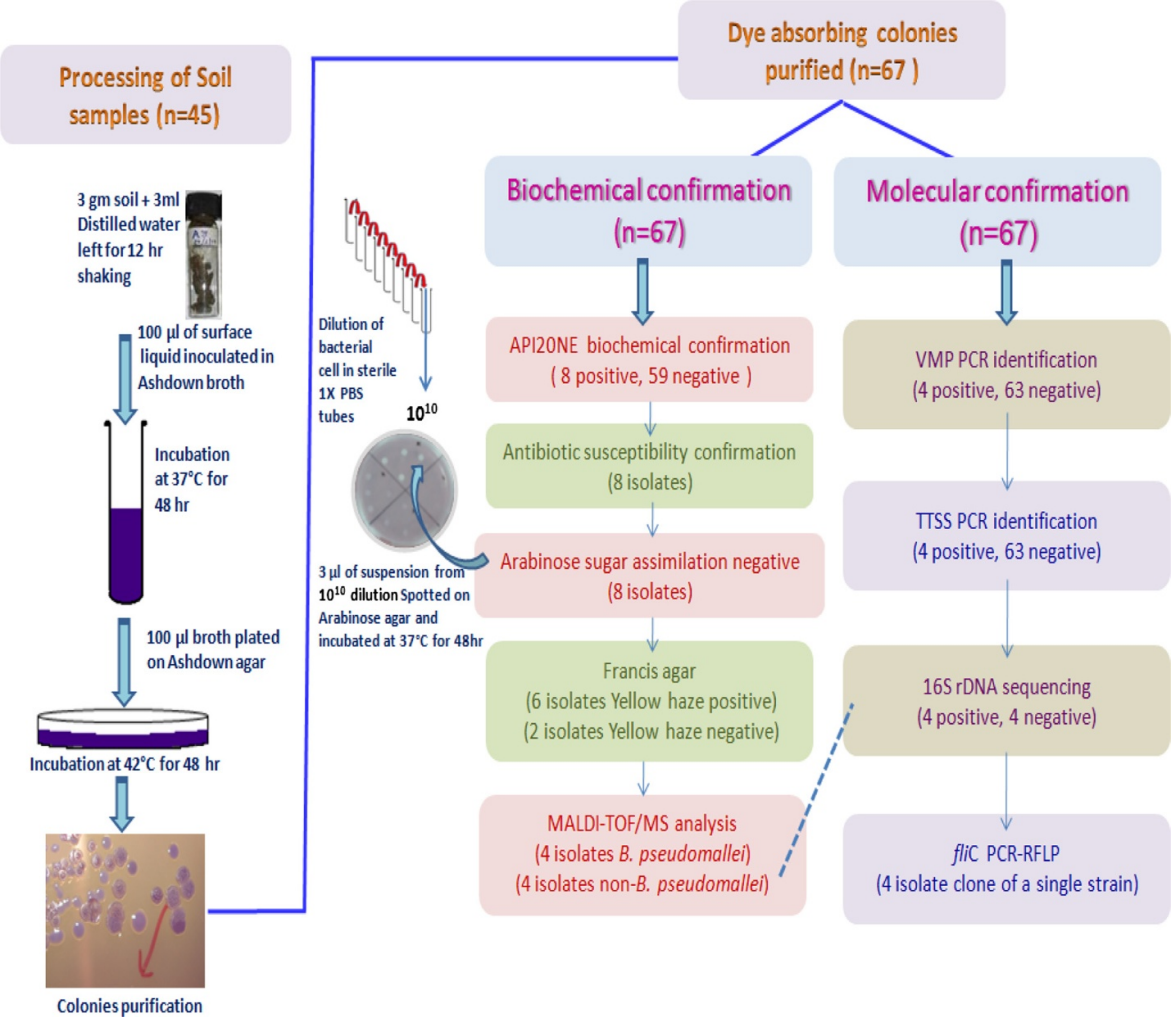
**Schematic presentation of isolation and identification procedure used in study.**

The soil samples collected were processed for the isolation of *Burkholderia pseudomallei* as per the following protocol. Briefly 3 gm of each soil sample was vigorously mixed with 3 ml of sterile distilled water and left for overnight. 100 μl of the upper surface liquid was then transferred into 5 ml Ashdown broth with a sterile pipette and incubated at 37°C for 48 hrs (Figure 2). Ashdown selective agar was modified from the recipe of Ashdown (1979) as follows: tryptone 1.5 g, glycerol 4 ml, crystal violet (25 mg/ml) 150μl, neutral red (25 mg/ml) 100 μl, gentamicin 8 μg/ml final concentration for 100 ml of medium. After incubation 100 μl of broth was plated onto Ashdown selective agar plates and incubated at 42°C. The plates incubated for four days were visually inspected daily until typical colonies formed as previously described (Chantratita et al., 2007). The colonies were purified by further subculture on Ashdown agar to confirm the purity and preserved in 30% glycerol stock at −20°C until further use.

### Biochemical and phenotypic confirmation

Initial screening of isolates was performed according to the standard protocols followed for the identification of *B. pseudomallei* (Sentinel Laboratory guidelines, 2003). Two standard strains, NCTC 1688 and NCTC 10274, were used as reference type strains along with the isolates. The commercially available API 20NE (Biomerieux) was also used for the generation of biochemical profiles of all soil isolates along with standard strain (Dance et al., 1989; Amornchai et al., 2007). Results were recorded after incubation of 24 hr to 48 hr at 28°C and interpreted referring to the data interpretation table from the API 20NE manual. *In vitro* antibiotic susceptibility of isolates for polymyxin B (100 units/disc) and colistin (25 mcg/disc) were also tested on Mueller–Hinton agar by the Kirby Bauer disk diffusion method (CLSI, 2007). Assimilation of L-arabinose was also tested conventionally as because it plays a key role in the discrimination of virulent *B. pseudomallei* from the nonvirulent species *B. thailandensis* (Wuthiekanum et al., 1996). The suspected isolates were further screened for yellow haze production on Francis agar for the discrimination of *B. pseudomallei* from *B. cepacia* (Francis et al., 2006).

### Molecular confirmation

#### Identification by Specific PCR

All eight biochemically identified *B. pseudomallei* isolates and 59 non *B. pseudomallei* isolates were further identified by PCR procedures based on amplification of 23S rDNA gene and the putative virulent determinant TTSS gene (Table 1). PCR was standardized with forward and reverse PCR primers and performed in a volume of 25 μl, the reaction mixture containing 200 mM of each dNTP, 1.5 mM MgCl_2_, 1 × PCR buffer, 10 pmol of each primer, 1 U of Taq DNA polymerase (Fermentas) and 10 ng DNA. The PCR cycle protocol consist of initial denaturation at 95°C for 6 min and 30 cycles of denaturation at 95°C for 1 min, primer specific annealing for 1 min and extension at 72°C for 2 min with the final extension at 72°C for 10 min. PCR products were electrophoresed on 1% agarose gel and visualised under UV in an Alpha Innotech Gel Imager (Amersham Pharmacia Biotech).

**Table 1:** **Primers used in molecular confirmation study**

Primer name	Primer sequences	Annealing temperature	Amplification size	Purpose/Reference
**VMP 23-1-F**	5′ CTTTTGGGTCATCCTRGA 3′	48°C	1,051 bp	Identification (Bauernfeind et al., 1998)
**MP 23-2-R**	5′ TCCTACCATGCGAGACT 3′			
**BPTTS1-F**	5′CGTCTCTATACTGTCGAGCAATCG 3′	58°C	548 bp	Identification (Novak et al., 2006)
**BPTTS1-R**	5′ CGTGCACACCGGTCAGTATC 3′			
**F8**	5′ AGTTTGATCCTGGCTCAG 3′	50°C	1,488 bp	16S sequencing (Gee et al., 2003)
**R1492**	5′ ACCTTGTTACGACTT 3′			
***fli*** **C-F**	5′ CTCGGATCCAACAGCAAC 3′	52°C	1,167 bp	PCR-RFLP (Primer designed)
***fli*** **C-R**	R- 5′ TATTGCAGGTACCTTCAG 3′			

#### 16S rDNA based phylogenic analysis

16S rDNA sequencing was used to confirm PCR identified isolates and 16S rDNA sequence of each isolate was BLAST analysed (Gee et al., 2003). The PCR reaction mixture for the amplification of the 16S rDNA gene consisted of 200 mM of each dNTP, 1.5 mM MgCl_2_, 1 × PCR buffer, 10 pmol of each primer, 1 U of Taq DNA polymerase (Fermentas) and 10 ng DNA. The reaction was made up to 25 μl with sterile distilled water and the cycle consisted of initial denaturation at 95°C for 6 min and 30 cycles of denaturation at 95°C for 1 min, annealing at 50°C for 1 min and extension at 72°C for 2 min with the final extension at 72°C for 10 min. PCR products were electrophoresed on 1.0% agarose gel and visualised under UV in a gel documentation system as above. Amplified 16S rDNA PCR products were sequenced by the dideoxy chain termination method using the Big dye Terminator v 3.1 cycle sequencing kit and Big dye X Terminator Purification kit in an ABI 10 sequencer (Applied Biosystems). The derived sequences were aligned using DNASTAR lasergene 9 Core Suit and BLAST analysis was performed for comparison with other bacterial species, sequences available in the NCBI database. A dendogram based on 16S rDNA sequence was also constructed by the neighbor joining method using Molecular Evolutionary Genetics Analysis version 5.0 (MEGA 5) analytical software (Tamura et al., 2011).

#### MALDI-TOF analysis for bacterial identification

Eight biochemically identified isolates were also MALDI-TOF/MS analyzed for the confirmation (Table 2). For the MALDI-TOF/MS analysis, purified single colonies from a 24 hr culture of each isolate was directly deposited on a MALDI-TOF MTP 96 target plate (Bruker Daltonik GmbH), in duplicate and 1 μl of 96% formic acid to each spot to inactivate the culture. The preparation was then overlaid with 1 μl of matrix solution that contained a saturated solution of α-cyano-4-hydroxycinnamic acid in 50% acetonitrile, and 2.5% trifluoroacetic acid. A total of 18 spots, two spots for the *E. coli* standard and 8 × 2 for the eight isolates were made on target plate. This matrix-sample was crystallized by air-drying at room temperature for 5 minutes. Measurements were performed with a Microflex III mass spectrometer (Bruker Daltonik) equipped with a 337- nm nitrogen laser. Spectra were recorded in the positive linear mode (delay, 170 ns; ion source 1 voltage, 20 kV; ion source 2 voltage, 18.5 kV; lens voltage, 6 kV; mass range, 2–20 kDa). Each spectrum was obtained after 240 shots in automatic mode at a variable laser power, and the acquisition time ranged from 30 to 60 s per spot. Data were automatically acquired using AutoXecute method of flexControl version 3.3 acquisition control software. The 2 first raw spectra obtained for each isolate were imported into Bio Typer software, version 3.0 (Bruker Daltonik GmbH), and were analyzed by standard pattern matching against the 4110 mass spectra of different bacterial species as reference from the Biotyper database.

**Table 2:** **Biochemical profile of standard strains and confirmed soil isolates**

Substrate	Standard strains		Soil isolates			
	***Burkholderia pseudomallei***NTCC10274	***Burkholderia pseudomallei***NTCC1688	DRDEBPS 1001	DRDEBPS 1002	DRDEBPS 1003	DRDEBPS 1004
**Nitrate**	+	+	+	+	+	+
**Tryptophan**	+	+	+	+	+	+
**Glucose (acidification)**	+	+	+	+	+	+
**Arginine**	+	+	+	+	+	+
Urea*	-	-	-	-	-	-
Esculin*	-	-	+	+	+	+
**Gelatin**	+	+	+	+	+	+
**PNPG**	-	-	-	-	-	-
**Assimilation of**						
**Glucose**	+	+	+	+	+	+
**Arabinose**	-	-	-	-	-	-
**Mannose**	+	+	+	+	+	+
**Mannitol**	+	+	+	+	+	+
**N-Acetyl glucosamine**	+	+	+	+	+	+
**Maltose**	-	-	-	-	-	-
**Gluconate**	+	+	+	+	+	+
**Caprate**	+	+	+	+	+	+
**Adipate**	+	+	+	+	+	+
**Malate**	+	+	+	+	+	+
**Citrate**	+	+	+	+	+	+
**Phenyl acetate**	+	+	+	+	+	+

*Variable tests for *B. pseudomallei.*

#### *Flagellin*C *(fli*C*) gene based restriction fragment length analysis*

Restriction fragment length analysis was performed on the specific PCR, 16S rDNA sequencing and MALDI-TOF/MS confirmed isolates to observe that whether these isolates belongs to similar strains (clone of a single strain) or belongs to different strains. The primers *fli*C forward 5′-CTC GGA TCCAAC AGC AAC-3′ and *fli*C reverse 5′-TAT TGC AGG TAC CTT CAG-3′ were used for the amplification of the 1167 bp *fli*C gene. The PCR amplification was carried out in a 50 μl reaction volume with the following PCR conditions: initial denaturation at 95°C for 6 min and 30 cycles of denaturation at 95°C for 1 min, annealing at 52°C for 1 min and extension at 72°C for 2 min with the final extension at 72°C for 10 min. PCR products were purified using a gel extraction purification kit (Qiagen) and concentration was determined spectrophotometrically. Restriction digestion was performed with three restriction enzymes *Dde* I, *Msp* I and *Pst* I (Fermentas, fast digest). Restriction digestion reaction mixture contained 7 μl of purified *fli*C PCR products at a conc. of 100 ng/μl and 1U of restriction enzymes. Restriction digestion reaction was carried out in 0.5 ml tubes incubated at 37°C for 2 hrs and then inactivated by keeping tubes in a dry bath at 65°C for 10 minutes. Then digested products were electrophoresed on 1.8% agarose gel and visualized under UV in an Alpha Innotech Gel Imager.

## Results & Discussion

From the 45 soil samples 67 isolates were suspected and selected as possible *B. pseudomallei* based on characteristic colonial morphology and dye absorption on Ashdown agar after incubation of 48 hr at 42°C. Conventional biochemical profiles of each of these 67 isolates was tested and compared with the profiles of *B. pseudomallei* NCTC 1688 and NCTC 10274. The biochemical profiles of eight isolates, namely DRDEBPS1001, DRDEBPS1002, DRDEBPS1003, DRDEBPS1004, DRDEBPS1018, DRDEBPS1019, DRDEBPS1020 and DRDEBPS1021 matched with the biochemical profile of *B. pseudomallei* with negative arabinose sugar assimilation. Interestingly all the environmental isolates were able to assimilate maltose but the standard strains could not assimilate this sugar, as also observed with most clinical isolates of *B. pseudomallei*. The biochemical profile of all these 67 isolates was determined by API 20NE and the eight isolates that were identified as *B. pseudomallei* by conventional biochemical assays were also positive by API 20NE. The identification of the remaining 59 isolates by API 20NE is given in Table 3. The antibiotic susceptibility of the 8 isolates that are biochemically consistent with *B. pseudomallei* was tested against antibiotics, polymyxin (B100 units/disc) and colistin (25 mcg/disc) and the susceptibility pattern was found similar to that of the *B. pseudomallei* standard strains.

**Table 3:** **Biochemical profiles of Ashdown grown suspected soil isolates confirmed by API20NE**

Species name	NO _3_/N _2_	TRP	GLU	ADH	URE	ESC	GEL	PNG	GLU	ARA	MNE	MAN	NAG	MAL	GNT	CAP	ADI	MLT	CIT	PAC
***Ochrobactrum anthropi (n = 3)***	+/+	-	-	-	+	-	-	-	+	+	+	-	+	+	+	+	-	+	-	-
***Achromobacter xylosoxidans (n = 5)***	+/+	-	-	-	-	-	-	-	-	-	-	-	+	-	+	+	+	+	+	+
***Chromobacterium violaceum (n = 9)***	+/+	-	+	+	-	-	+	-	+	-	+	+	+	-	+	+	+	+	+	+
***Stenotrophomonas maltophilia (n = 10)***	+/+	-	-	-	-	+	+	+	+	-	+	-	+	+	-	-	-	+	+	-
***Rizobium radiobacter (n = 1)***	+/+	-	-	-	+	+	-	+	+	+	+	+	+	+	+	-	-	+	+	-
***Burkholderia cepacia (n = 4)***	+/+	-	+	-	-	+	+	+	+	+	+	+	+	-	+	+	-	+	+	+
***Delftia acidovorans (n = 5)***	+/−	-	-	-	-	-	-	-	-	-	-	+	-	-	+	+	+	+	+	+
***Ralstonia pickettii (n = 3)***	-	-	-	-	-	-	-	-	+	-	-	-	-	-	+	+	+	+	+	-
***Enterococcus faecalis (n = 2)***	+/+	-	+	-	-	+	-	+	-	-	-	-	-	-	-	+	-	-	+	-
***Brevundimonas vesicularis (n = 1)***	**-**	-	-	-	-	+	-	-	+	-	-	-	-	+	-	-	-	-	-	-
***Pseudomonas luteola (n = 2)***	+/+	-	-	+	-	+	-	+	+	+	+	+	-	+	+	+	-	+	+	-
***Comamonas testosterone (n = 1)***	+/+	-	-	-	-	-	-	-	-	-	-	-	-	-	-	+	-	+	-	-
***Pseudomonas aeruginosa (n = 5)***	+/+	-	-	+	+	-	+	-	+	-	-	+	+	-	+	+	+	+	+	-
***Acinetobacter lwoffii (n = 1)***	-	-	-	-	-	-	-	-	-	-	-	-	-	-	-	+	-	+	-	+
***Acinetobacter dentrificans (n = 2)***	+/+	-	-	-	-	-	-	-	-	-	-	-	-	-	+	-	+	+	+	+
***Pseudomonas mendocina (n = 1)***	+/+	-	-	+	-	-	-	-	+	-	-	-	-	-	+	+	-	+	+	-
***Chryseobacterium indologenens(n = 4)***	+/+	+	-	-	+	+	+	-	+	-	-	-	-	+	-	-	-	-	-	-

**Figure 3:**
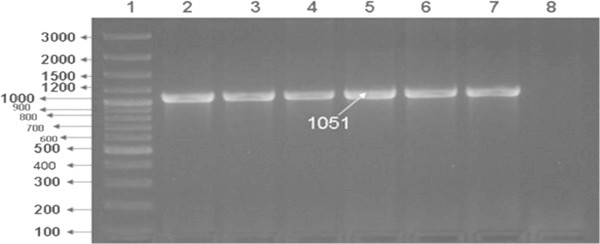
**VMP PCR for the confirmation of isolates- Lane1: 100 bp plus Marker, Lane 2:** ***B. pseudomallei*** **NTCC 10274, Lane 3:** ***B. pseudomallei*** **NTCC 1688, Lane 4: Soil isolate DRDEBPS1001, Lane 5: DRDEBPS1002, Lane 6: DRDEBPS1003, Lane 7: DRDEBPS1004, Lane 8: Negative control.**

**Figure 4:**
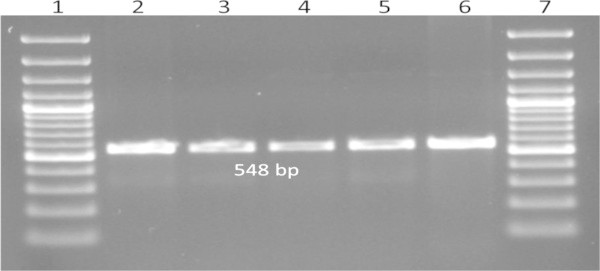
**TTSS PCR for the confirmation of isolates- Lane1: 100 bp plus Marker, Lane 2:** ***B. pseudomallei*** **NTCC 10274, Lane 3: Soil isolate DRDEBPS1001, Lane 4: DRDEBPS1002, Lane 5: DRDEBPS1003, Lane 6: DRDEBPS1004, Lane 7: 100 bp plus Marker.**

**Figure 5:**
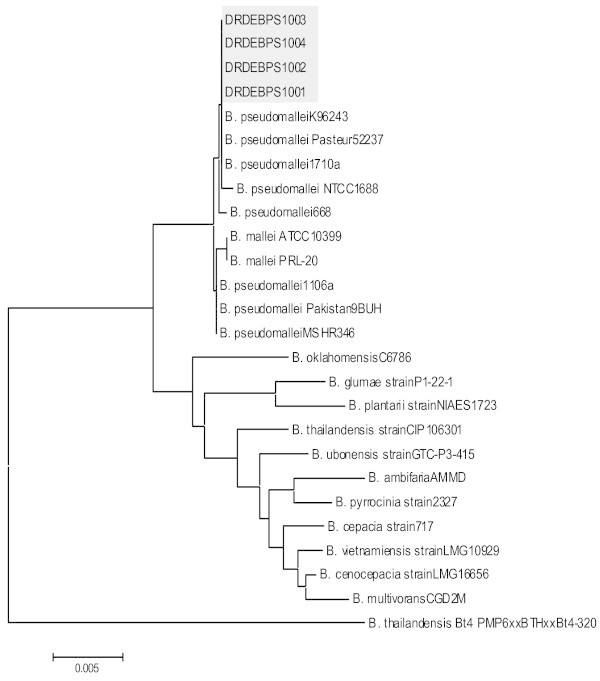
**Dendogram based on 16S rDNA sequencing constructed by neighbor joining method (Bar represents 0.005 substitution per site).** Soil isolates DRDEBPS1001, DRDEBPS1002, DRDEBPS1003 and DRDEBPS1004 showing proximity to *B. pseudomallei* standard strains.

These eight isolates were further processed for molecular confirmation and only four DRDEBPS1001, DRDEBPS1002, DRDEBPS1003 and DRDEBPS1004 were found positive by *B. pseudomallei* specific PCR for the truncated region of 23S rDNA (VMP PCR) and TTSS gene (TTSS PCR) with the amplification of 1,051 bp and 548 bp respectively (Figures 3 and 4). Both these PCRs were also performed with the DNA of 59 isolates that were biochemically grouped as non *B. pseudomallei* and no amplification was observed in these isolates confirming the biochemical results. Sequencing of 16S rDNA of these eight isolates confirmed that, the four isolates showed 100% homology with the NCBI database reference strains of *B. pseudomallei.* Isolates DRDEBPS1018, DRDEBPS1019 were identified as *Cupriavidus necator* and isolates DRDEBPS1020, DRDEBPS1021 as *Enterobacter cloacae*. The aligned partial sequences of 16S rDNA were submitted to GenBank and accession numbers JN001986, JN001987, JN001988 and JN001989 were obtained. A phylogenetic tree based on 16S rDNA sequences of all four confirmed isolates was also constructed along with the standard strains of *B. pseudomallei* and other closely related species (Figure 5). The phylogenetic analysis also revealed that all four isolates closely matched with the standard strains of *B. pseudomallei* and were placed in the same clade.

**Figure 6:**
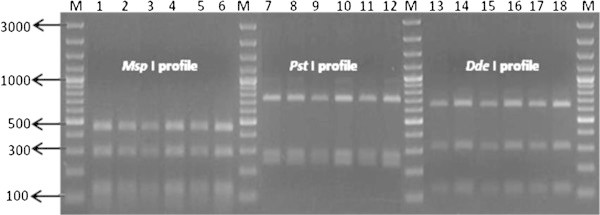
***fli*** **C gene Restriction patterns of isolates Lane: M 100 bp plus Marker, Lane 1, 7, 13:** ***B. pseudomallei*** **NTCC 10274, Lane 2, 8, 14:** ***B. pseudomallei*** **NTCC 1688, Lane 3, 9, 15: Soil isolate DRDEBPS1001, Lane 4, 10, 16: DRDEBPS1002, Lane 5, 11, 17: Soil isolate DRDEBPS1003, Lane 6,12,18: Soil isolate DRDEBPS1004.**

The four isolates confirmed by 16S rDNA sequencing were subjected to PCR-RFLP for the *fli*C locus and restriction profiles for three different restriction enzymes were generated. The restriction profile after digestion with *Msp* I produced 443, 285, 134, 123, 99, 64, 19 bp size products, *Pst* I digestion produced 681, 266, 220 bp size products and *Dde* I enzyme digestion produced 628, 307, 123, 109 bp size products and the restriction profile for all these three enzymes matched with *B. pseudomallei* standard strains NCTC 1688 and NCTC 10274, thereby confirming all four isolates as *B. pseudomallei* with identical restriction profiles (Figure 6).

Recently MALDI-TOF mass spectrum analysis has been considered an easy and discriminatory tool for identification of bacterial species (Lista et al., 2011). The results of MALDI-TOF mass spectrum analysis of the eight suspected isolates matched with that of the 16S rDNA sequence analysis. The four isolates DRDEBPS1001, DRDEBPS1002, DRDEBPS1003 and DRDEBPS1004 were confirmed as *B. pseudomallei* on the basis of score values 2.601, 2.099, 2.362 and 2.047. The other four isolates had been biochemically suspected but not supported by both the PCRs were also identified by MALDI-TOF spectrum analysis as *Cupriavidus necator* and *Enterobacter cloacae* with score value of 2.112, 2.122 and 2.341, 2.241 respectively, confirming the results of the 16S analysis.

The isolation of *B. pseudomallei* from soil is very complex as the presence of large numbers of closely related soil microflora interferes with its recovery although use of Ashdown broth and agar for the isolation of *B. pseudomallei* from soil samples provides some selectivity. This selectivity comes from crystal violet, neutral red and gentamicin present in the Ashdown medium. The vast majority of *B. pseudomallei* isolates are resistant to aminoglycosides due to expression of a multidrug efflux pump, thus allowing use of gentamicin for selection. This study confirms that, although this medium is useful for initial screening, many other species also grow on this medium thus interfering the recovery of *B. pseudomallei*. Francis agar is a medium that can clearly differentiate between *B. pseudomallei* and other closely related species including *B. cepacia* (Francis et al., 2006). We also analyzed all 8 suspected isolates of *B. pseudomallei* on Francis agar to observe the yellow haze around colonies 24 hr. Although Francis Agar was not developed for primary soil isolation of this species it can be used to confirm suspected colonies isolated from Ashdown agar. In our study the rate of isolation was found to be 5.97% as 4 isolates were confirmed as *B. pseudomallei* out of 67 suspected isolates from Ashdown agar and 2 isolates each were obtained from site 1 and site 2 (Figure 1). In similar soil isolation studies conducted in Malaysia and Thailand, high isolation rates were found in wet rice fields and other cleared and cultivated areas (Brett et al., 1998; Smith et al., 1995). This study reports the first isolation of *B. pseudomallei* from soil collected from paddy fields in the coastal region of India. The study site selected in this study fulfilled with all criteria of soil sampling which were considered as important factors in the isolation of *B. pseudomallei*. The presence of *B. pseudomallei* depends on various environmental factors that support the survival of *B. pseudomallei* in soil. The presence of *B. pseudomallei* in soil is high during when ploughing and planting of seedlings takes place (Palasatien et al., 2008; Currie et al., 2004). The maintenance of viable bacteria in soil samples during collection, transport and storage before processing in laboratory is also an important factor for the isolation. The presence of *B. pseudomallei* needs to be initially determined by culture based methods in microbiological laboratories that require only basic equipment and provides live organisms for further confirmation by DNA based molecular methods. The isolates recovered from soil need to be compared with those recovered from clinical cases as it provides vital information on the pathogenicity and virulence of soil isolates (Currie et al., 2004). The presence of identical genetic patterns among clinical and environmental isolates suggests a link between the bacteria present in contaminated soil and the emergence of indigenous melioidosis (Chen et al., 2010). Future studies are also recommended with large number of samples collected from different geographical regions of India to study the pattern of distribution of this important bacterial species and also to correlate the epidemiological relevance of soil isolation to the occurrence of melioidosis.

## Conclusion

We report the first isolation and molecular confirmation of *B. pseudomallei* from soil of paddy fields in the coastal region of India. These isolates were initially identified by conventional biochemical methods and further confirmed by advanced molecular based methods of 16S rDNA sequencing, *B. pseudomallei* specific PCR, PCR-RFLP at *fli*C locus and MALDI-TOF based protein profiling. This study confirms the presence of *B. pseudomallei* in soil in the coastal region of India. The isolation of this important bacterial species from this part of India should initiate further studies on the extent of environmental and clinical impact of melioidosis in India.
